# Evaluation of childhood traumatic experience as a risk factor for alcohol use disorder in adulthood

**DOI:** 10.1186/s12888-020-2428-5

**Published:** 2020-01-09

**Authors:** Lan Wang, Cui-Xia An, Mei Song, Na Li, Yuan-Yuan Gao, Xiao-Chuan Zhao, Lu-Lu Yu, Yu-Mei Wang, Xue-Yi Wang

**Affiliations:** 1 Department of Psychiatry, The First Hospital of Hebei Medical University, No. 89 Donggang Road, Shijiazhuang, 050031 China; 2 The Mental Health Institute of Hebei Medical University, Shijiazhuang, China; 3 Hebei Brain Ageing and Cognitive Neuroscience Laboratory, Shijiazhuang, China

**Keywords:** Childhood trauma, Alcohol use disorder, Earthquake stress, Risk factor

## Abstract

**Background:**

We aimed to investigate the effect of early-age (prenatal, infant, and childhood) trauma on adulthood alcohol use disorder.

**Methods:**

A total number of 1534 subjects who were born and live in the city of Tangshan were selected. The subjects were divided into three age groups. General demographic data, conditions of the mothers during pregnancy, and condition of the babies at birth, were collected. The diagnosis of alcohol use disorder was based on Structured Clinical Interviews for DSM-IV Axis Disorders (patient version) (SCID). The childhood trauma questionnaire short form (CTQ-SF) [1] and the Lifetime of Experience Questionnaire (LTE-Q) [2] were used to evaluate stress in childhood and adulthood, respectively.

**Results:**

Only male subjects were diagnosed with lifelong alcohol abuse and alcohol dependence. There was no statistically significant difference in the prevalence of lifetime alcohol use disorder (*X*^*2*^ = 4.480, *P* = 0.345), current alcohol abuse, and current alcohol dependence among the three groups (*X*^*2*^_*abuse*_ = 2.177, *X*^*2*^_*depedence*_ = 2.198, *P* > 0.05). However, higher prevalence of lifetime alcohol use disorders was found in group with higher scores of CTQ (*X*^*2*^ = 9.315, *P* = 0.009), emotional abuse (*X*^*2*^ = 8.025, *P* = 0.018), physical abuse (*X*^*2*^ = 20.4080, *P* < 0.001), but not in the group with higher scores of emotional neglect (*X*^*2*^ = 1.226, *P* = 0.542), sexual abuse (*X*^*2*^ = 2.779, *P* = 0.249), physical neglect (*X*^*2*^ = 3.978, *P* = 0.137), LTE-Q (*X*^*2*^ = 5.415, *P* = 0.067), and PSQI (*X*^*2*^ = 5.238, *P* = 0.073). Protective factor for alcohol abuse for men was identified to be heavy drinking (OR = 0.085, 95%CI: 0.011–0.661), and the risk factors for alcohol abuse were identified to be frequent drinking (OR = 2.736, 95%CI: 1.500, 4.988), and consumption of low liquor (OR = 2.563, 95%CI: 1.387, 4.734). Risk factors for alcohol dependence in males were identified to be consumption of low liquor (OR = 5.501, 95%CI: 2.004, 15.103), frequent drinking (OR = 2.680, 95%CI: 1.164, 6.170), and childhood physical abuse (OR = 2.310, 95% CI: 1.026, 5.201).

**Conclusion:**

Traumatic experience during infant and prenatal periods does not have a strong statistical correlation with alcohol use disorders for male adults. However, subjects with high CTQ scores, experience of emotional abuse and physical abuse show a statistically higher prevalence of lifetime alcohol use disorders. Several risk factors including consumption of low liquor, frequent drinking, and childhood physical abuse contribute to alcohol dependence in male adults.

## Background

Alcohol dependence is a growing social epidemic around the world and it poses a significant threat to the well being of the affected individuals and the entire society [3, 4]. Specifically, alcohol addiction has not only led to a marked increase in social issues such as violent crime, traffic accidents and soaring divorce rates, but also caused a variety of psychological and physical diseases, producing a heavy burden on the health care system [5]. With the largest population in the world, China has witnessed a rapid growth of alcohol dependence prevalence from 0.4% in 1985 to 9% in 2009 [6]. The growing prevalence of alcohol dependence has attracted attention from scientific community seeking to unveil the underlying mechanism of alcohol dependence and address the need of effective alcohol addiction therapy [7]. The mechanism of alcohol use disorder is complicated and increasing evidence showed that stress is an important factor implicated in the pathological mechanism of alcohol dependence [8, 9]. For example, Noori and Yu have performed rats foot shock and forced swim stress studies, showing stress can lead to increased alcohol intake and alcohol conditioned place preference [ 10,11]. In addition to the stress in adulthood, early life stress (ELS) is found to be related to alcohol dependence as well. Marinelli and co-workers confirmed that stress during the prenatal period is correlated to increased addiction risk during adulthood [12]. Further, it was found that maternal separation (MS) - a form of ELS - is a risk factor for binge drinking, and is linked to impulsivity, another key risk factor for excessive alcohol drinking in adulthood [13]. People with a history of childhood maltreatment, such as those in a war-exposed region for more than 30 days, showed 5.3 times higher chance of subsequent alcohol disorders compared to those not exposed [14]. It was hypothesized that the experience of ELS caused long-lasting modulation of neurons, as well as hyperactivity of the hypothalamuspituitary-adrenal (HPA) axis [15]. However, most research on the relationship between trauma and alcohol use disorders focus on a single age group, e.g., trauma happened during either childhood or adulthood [16, 17]. Furthermore, most of the previous studies on prenatal period stress are animal studies [18]. To the best of our knowledge, there are no reports on how the same ELS event affects individuals from different life stages (i.e., prenatal, infant, or childhood) and the direct comparison of the effect of a single ELS event on adulthood alcohol use disorder for different age groups. Therefore, we report herein our studies on the effects of a series of contributing factors - the 1976 Tangshan Earthquake, childhood traumatic experience, adulthood traumatic experience, and sleeping quality - on adulthood alcohol use disorder. We also aim to study the effects of Earthquake on alcohol use disorder for subjects who experienced the Earthquake at different stages of pregnancy (prenatal and infant) and present our analysis of the risk factors for adulthood alcohol use disorder with the same group of subjects.

## Methods

### Subjects

The subjects of this study were recruited from workers officially employed by the Kailuan Mining Group. Based on the dates of birth, the subjects were divided into three groups (from the eldest to the youngest): infant exposure group, prenatal exposure group, and non-exposure group.

The inclusion criteria for the infant exposure group included: 1) born and raised in Tangshan, 2) born between July 29, 1975 and April 28, 1976; 3) exposure to the earthquake at the age between 3 and 12 months.

The inclusion criteria for the prenatal exposure group included: 1) born and raised in Tangshan; 2) born between July 29, 1976 and April 28, 1977; 3) exposure to the earthquake during the prenatal period. Based on the age of the fetal when the mother was exposed to earthquake, this group was further divided into 3 subgroups: the first trimester group (1–3 months of mother’s pregnancy during the earthquake), the second trimester group (4–6 months of mother’s pregnancy during the earthquake), and the third trimester group (7–9 months of mother’s pregnancy during the earthquake).

The inclusion criteria for the non-exposure group included: 1) born and raised in Tangshan; 2) born between July 29, 1977 and April 28, 1978; and 3) born 1–1.9 years after the earthquake.

The following exclusion criteria were applied: 1) mothers had infections, high blood pressure, epilepsy or seizures, diabetes, thyroid disease, or mental disorders during pregnancy [2]; mothers had history of drug use, drinking and poisoning during maternal or lactating period [3]; mother suffered from other traumatic events in addition to earthquake during pregnancy [4]; those who refused to participate in this study and did not sign the informed consent form.

### Methods

This study is a cross-sectional study [19] and is approved by the Ethics Committee of the First Hospital of Hebei Medical University (No. 2014005). The research was registered at Chinese Clinical Trial Registry (No. ChiCTR-OOC-15006542). From January to December of 2014, 38 years after the earthquake, a total of 1534 eligible participants were recruited from Kailuan Mining Group. A total of 1325 subjects completed the study with a completion rate of 85.9%. Each of the participants signed a written informed consent form. Standardized interviews and physical examinations are conducted by specially trained doctors in one-on-one interviews.

#### Collection of demographic information

In brief, all participants underwent a physical examination and a standardized interview, which included questions about demographic information, the condition of mother during pregnancy (age at pregnancy, parity, etc.), the conditions of the babies (weight at birth), and smoking history, and family history of alcohol use disorder.

#### Evaluation of alcohol use disorders

Structured Clinical Interviews for DSM-IV Axis Disorders (patient version) (SCID) were performed on all subjects by psychiatrists for the diagnosis of alcohol use disorders according to literature protcols [20].

In addition to the diagnosis of lifetime/current alcohol dependence and lifetime/current alcohol abuse, the following information was also collected: self-reported use of alcohol, including frequency of drinking: drinking often currently (≥2 times/week), used to drink often but do not drink currently (less than 1 time/year), drinking occasionally (< 2 times/year), and no drinking history (≤1 time/year); amount of alcohol consumed (according to the average daily alcohol consumption for men, binge is defined as drinking more than 90 mL of pure alcohol/day, heavy drinking is defined as drinking more than 50 mL but less than 90 mL of alcohol/day, normal drinking is defined as drinking less than 50 mL of pure alcohol/day); time of drinking; type of alcohol consumed: beer, red wine, low liquor (< 40% volume), high liquor (≥40% volume).

#### Childhood trauma

Childhood trauma was assessed by childhood trauma questionnaire short form (CTQ-SF) [21], Chinese version, which is a retrospective self-report measurement with five dimensions: emotional abuse, physical abuse, sexual abuse, emotional neglect, and physical neglect. The scores obtained from five different dimensions were used to assess childhood experiences. The CTQ-SF has 28 entries in total, including 25 clinical items and 3 validation items. Each question starts with the sentence of “When I grew up and before I was 16 years old.” The 20th question, for instance, was followed by, “somebody attempted to touch me or let me touch him in a sexual way.” Based on the frequency of occurrence, the answer may be scored as follows: 1 point, never; 2 points, occasionally; 3 points, sometimes; 4 points, regular; 5 points, always. In the present study, for each dimension, the subtotal score was the sum of the score of each item that falls within that respective dimension, and the total score was the sum of the scores of the five dimensions [22]. Each of the five dimensions listed above is scored between 5 to 25 points, with a total score between 25 to 125 points.

#### Adult trauma

Adult trauma beginning at the age of 16 was evaluated by Lifetime of Experience Questionnaire (LTE-Q) [2]. With minor modifications to adapt to special circumstances in China, this questionnaire included questions on the following items [1]: loss of a spouse [2]; loss of parents and children [3]; economic difficulties [4]; divorce [5]; unemployment or layoff [6]; accidents (traffic accidents, fires, flooding, earthquake, or other natural disasters) [7]; incidents related to the crimes such as property loss, robbery, or kidnap [8]; others.

#### Sleep quality

Pittsburgh Sleep Quality Index (PSQI) [23], Chinese version, was calculated for the evaluation of sleep quality. The PSQI is composed of 19 self-assessments and 5 review questions, and only 18 self-assessment questions are scored. The sum of the score for each component is the PSQI total score, ranging from 0 to 2 l. In general, score ≥ 7 indicates sleep problems [24].

### Statistical analysis

IBM SPSS version 22.0 was used for statistical analysis. The data is represented as the mean ± standard deviation (SD). Chi-square tests were performed to compare the prevalence of alcohol use disorders among subjects categorized by various contributing factors listed above. Stepwise regression with forward selection approach was employed for method of analysis of all contributing factors. One-way ANOVA of variance or rank-sum test with multiple samples were performed for analysis of age and weight at birth. Multi-factor logistic regression was used to analyze risk factors for alcohol use disorders. A two-sided *P*-value < 0.05 was considered statistically significant.

## Results

### Baseline characteristics of study subjects

**Fig. 1:**
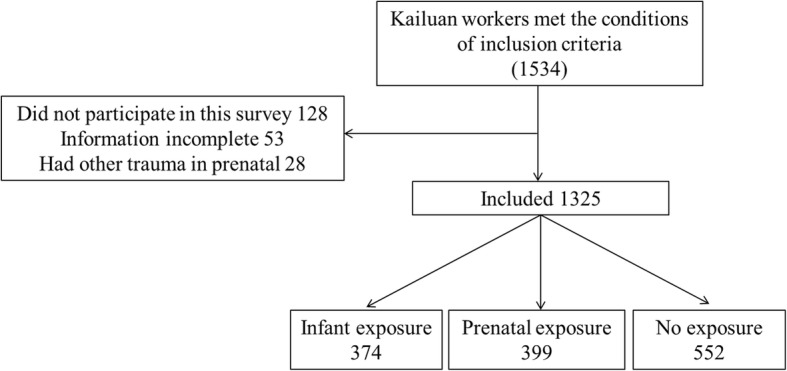
The study follow chart

1534 subjects met the inclusion criteria, but 129 of them did not participate in this study. As a result, we enrolled 1405 subjects for this study, of which 81 were excluded as shown in Fig. 1, leaving 1325 subjects on which statistical analysis was ultimately performed. As shown in Table 1, there is expected significant difference on the mean age of the three groups, namely, infant exposure, prenatal exposure, and non-exposure groups (*F* = 931.979, *P* < 0.001). In particular, the mean age of these three groups differ by one year in the decreasing order. However, no significant difference among the three age groups is observed for gender (*X*^*2*^ = 0.786, *P* = 0.675), education (*X*^*2*^ = 12.597, *P* = 0.050), marital status (*X*^*2*^ = 2.976, *P* = 0.812), smoke (*X*^*2*^ = 2.088, *P* = 0.719), mother’s age at birth (*F* = 4.211, *P* = 0.122), parity (*X*^*2*^ = 1.226, *P* = 0.874), birth weight (*F* = 0.066, *P* = 0.936), and family history of alcohol dependence (*X*^*2*^ = 0.147, *P* = 0.929).

**Table 1:** Baseline characteristics of the subjects (*N* = 1325)

		Infant exposure group	Prenatal exposure group	No exposure group	Test value	*P*
N		374	399	552		
Gender (%)	Male	317(84.8)	348(87.2)	475(86.1)	0.786	0.675
	Female	57(15.2)	51(12.8)	77(13.9)		
Age (year)		39.5 ± 0.6	38.5 ± 0.8	37.5 ± 0.9	931.979	<0.001
Education (%)	Illiterate and primary school	9(2.4)	8(2.0)	6(1.1)	12.597	0.05
	Junior high school	70(18.7)	64(16.0)	73(13.2)		
	High school / secondary school	197(52.7)	221(55.4)	289(52.4)		
	College and above	98(26.2)	106(26.6)	184(33.3)		
Marital status (%)	Unmarried	4(1.1)	7(1.8)	3(0.5)	2.976	0.812
	Married	351(93.9)	378(94.7)	518(93.8)		
	Divorced	16(4.3)	17(4.3)	29(5.3)		
	Widowed	3(0.8)	1(0.3)	2(0.4)		
Average household income (%)	≤1000 RMB	7(1.9)	15(3.8)	17(3.1)	7.397	0.494
	1001–2000 RMB	98(26.2)	97(24.3)	138(25.0)		
	2001-5000RMB	247(66.0)	251(62.9)	347(62.9)		
	5001–10,000 RMB	22(5.9)	34(8.5)	48(8.7)		
	>10,000 RMB	0	2(0.5)	2(0.4)		
Smoke (%)	smoker	182(48.7)	195(48.9)	280(50.7)	2.088	0.719
	ex-smoker	31(8.3)	34(8.5)	55(10.0)		
	Non-smoker	161(43.0)	170(42.6)	217(39.3)		
N		374	399	552		
Mother’s age at birth		27.2 ± 4.3	27.4 ± 4.9	27.7 ± 4.5	4.211	0.122
Parity (%)	First born	196(52.4)	208(52.1)	269(48.7)	1.226	0.874
	Second child	99(26.5)	110(27.6)	133(24.1)		
	Other	79(21.1)	81(20.3)	120(21.7)		
Birth weight (g)		3203.6 ± 571.5	3195.4 ± 593.8	3187.6 ± 570.9	0.066	0.936
Alcohol dependence family history (%)		10(2.7)	9(2.3)	14(2.5)	0.147	0.929

### The prevalence of alcohol use disorders

Since no diagnosis of lifetime alcohol abuse and alcohol dependence was observed for females, the following data analysis is based on male subjects only (See Table 2). Based on the results of chi-square test, there was no statistically significant difference among three age groups in the prevalence of subjects who are diagnosed with no alcohol use disorders (*X*^*2*^ = 4.480, *P* = 0.345), as well as in the prevalence of subjects who are diagnosed of current alcohol abuse (*X*^*2*^ = 2.177, *P* = 0.337) and current alcohol dependence (*X*^*2*^ = 2.098, *P* = 0.350). The prevalence of lifetime alcohol dependence in infant exposure group, prenatal exposure group and non-exposure group was 4.7, 3.2, 2.7% respectively. The prevalence of lifetime or current alcohol abuse was substantially higher in the non-exposure group than that of the two exposure groups.

**Table 2:** Prevalence of alcohol use disorder (*N* = 1325)

		Infant exposure group	Prenatal exposure group	Non-exposure group	Test value	*P*
N		317	348	475		
Alcohol use disorder for lifelong diagnostics (%)	No	288(90.9)	321(92.2)	431(90.7)	4.480	0.345
	Alcohol abuse	14(4.4)	16(4.6)	31(6.5)		
	Alcohol dependence	15(4.7)	11(3.2)	13(2.7)		
Alcohol use disorder for current diagnostics (%)	Alcohol abuse	6(1.2)	3(0.9)	11(2.3)	2.177	0.337
	Alcohol dependence	10(3.2)	9(2.6)	7(1.5)	2.098	0.350

Further, when considering the prenatal exposure group alone, it was found that there was no statistically significant difference in the prevalence of lifelong diagnosis of alcohol use disorders among three subgroups of prenatal exposure group (*X*^*2*^ = 1.136, *P* = 0.980). Similarly, there was not statistically significant difference in the prevalence of current diagnosis of alcohol abuse and dependence among three prenatal subgroups (*X*^*2*^_*abuse*_ = 3.773, *P*_*abuse*_ = 0.287; *X*^*2*^_*dependence*_ = 0.369, *P*_*dependence*_ = 0.947) (See Table 3).

**Table 3:** Prevalence of alcohol use disorder in different stages of pregnancy (*N* = 348)

		Early pregnancy	Mid- pregnancy	Late pregnancy	Test value	*P*
N		110	117	121		
Alcohol use disorder for lifelong diagnostics (%)	No	100(90.9)	108(92.3)	111(91.7)	1.136	0.980
	Alcohol abuse	5(4.5)	5(4.3)	6(5.0)		
	Alcohol dependence	5(4.5)	4(3.4)	4(3.3)		
Alcohol use disorder for current diagnostics (%)	Alcohol abuse	0	2(1.7)	1(0.8)	3.773	0.287
	Alcohol dependence	3(2.7)	3(2.6)	3(2.4)	0.369	0.947

### Childhood, adulthood trauma and sleep quality

According to the scores of CTQ, LTE-Q, and PSQI, the subjects were categorized as high and low score subgroups. After chi-square test, it was found there was statistically significant difference in the prevalence of lifetime alcohol use disorders between the subgroups with high and low scores in CTQ (*X*^*2*^ = 9.315, *P* = 0.009), emotional abuse (*X*^*2*^ = 8.025, *P* = 0.018), physical abuse (*X*^*2*^ = 20.4080, *P* < 0.001). However, no statistically significant difference was observed between the subgroups with high and low scores in emotional neglect (*X*^*2*^ = 1.226, *P* = 0.542), sexual abuse (*X*^*2*^ = 2.779, *P* = 0.249), physical neglect (*X*^*2*^ = 3.978, *P* = 0.137), LTE-Q (*X*^*2*^ = 5.415, *P* = 0.067), and PSQI total scores (*X*^*2*^ = 5.238, *P* = 0.073) (See Table 4).

**Table 4:** Prevalence of alcohol use disorder for male subjects with high and low scores of CTQ, LTE-Q, and PSQI (*N* = 1140)

		No	Alcohol abuse	Alcohol dependence	*X*^*2*^	*P*
N		1041	60	39		
CTQ total scores (%)^a^	High	369(88.7)	24(5.8)	23(5.5)	9.315	0.009
	Low	672(92.8)	36(5.0)	16(2.2)		
Emotional abuse (%)	High	320(88.4)	22 (6.1)	20(5.5)	8.025	0.018
	Low	721(92.7)	38(4.9)	19(2.4)		
Emotional neglect (%)	High	364(90.5)	21(5.2)	17(4.2)	1.226	0.542
	Low	677(91.7)	39(5.3)	22(3.0)		
Sexual abuse (%)	High	164(89.6)	9(4.9)	10(5.5)	2.779	0.249
	Low	877(91.6)	51(5.3)	29(3.0)		
Physical neglect (%)	High	415(89.4)	28(6.0)	21(4.5)	3.978	0.137
	Low	626 (92.6)	32(4.7)	18(2.7)		
Physical abuse (%)	High	185(85.3)	14(6.5)	18(8.3)	20.408	< 0.001
	Low	856(92.7)	46(5.0)	21(2.3)		
LTE-Q total scores (%)	High	392(90.7)	19(4.4)	21(4.9)	5.415	0.067
	Low	648(91.5)	42(5.9)	18(2.5)		
PQSI total scores (%)	High	171(87.2)	15(7.7)	10(5.1)	5.238	0.073
	Low	871(92.3)	45(4.8)	28(3.0)		

(a) Low score is defined as 25–75 and high score is defined as 76–125

### Drinking condition

As shown in Table 5, statistically significant difference on the prevalence of lifetime alcohol use disorders was observed with different wine categories (*X*^*2*^ = 34.446, *P* < 0.001), drinking frequency (*X*^*2*^ = 97.451, *P* < 0.001), drinking time (*X*^*2*^ = 10.438, *P* = 0.034), and alcohol consumption (*X*^*2*^ = 49.596, *P* < 0.001).

**Table 5:** Prevalence of alcohol use disorder for male subjects with different wine categories, drinking frequency, drinking years, and amount of alcohol consumption (*N* = 1140)

		No	Alcohol abuse	Alcohol dependence	Test value	*P*
N		1036	60	39		
Wine category (%)	Beer	543(94.6)	23(4.0)	8(1.4)	43.458	< 0.001
	Red wine	10(90.9)	1(9.1)	0		
	Low liquor	400(82.5)	46(9.5)	39(8.0)		
	High liquor	62(88.6)	5(7.1)	3(4.3)		
Drinking frequency (%)	Frequently	190(78.8)	29(12.0)	22(9.1)	72.604	< 0.001
	Quit	34 (89.5)	1(2.6)	3(7.9)		
	Occasionally	586(92.9)	31(4.9)	14(2.2)		
	No	230(99.6)	1(0.4)	0		
Year of drinking (%)	0–10	557(90.3)	41(6.6)	19(3.1)	13.775	0.008
	11–20	434(86.3)	37(7.4)	32 (6.4)		
	21–30	14(70.0)	4(20.0)	2 (10.0)		
Amount of alcohol consumption (%)	Normal	980(92.1)	57(5.4)	27(2.5)	40.428	< 0.001
	Heavy	49(80.3)	2(3.3)	10(16.4)		
	Binge	11(73.3)	2(13.3)	2(13.3)		

### Risk factors for alcohol use disorders

Logistic regression analysis was subsequently performed to identify risk factors for alcohol use disorders. As shown in Tables 6 and 7, heavy drinking (OR = 5.679, 95%CI: 1.576, 20.471) and frequency drinking (OR = 2.159, 95%CI: 1.136, 4.103) were identified to be the risk factors of alcohol abuse, and similar OR value after adjusting for age and education. For alcohol dependency, consumption of low liquor (OR = 5.342, 95%CI: 1.842, 15.495), frequent drinking (OR = 2.957, 95%CI: 1.222, 7.157), and childhood physical abuse (OR = 2.705, 95% CI: 1.303, 5.615) were identified as risk factors, and similar OR value after adjusting for age and education.

**Table 6:** Multi-factor logistic regression analysis of risk factor for alcohol abuse (*N* = 1140)

		B	SE	wald	*P*	OR	95%CI	aOR	*P′*	95%CI’
Wine category	High liquor	0.141	0.687	0.042	0.837	1.152	(0.300, 4.427)	1.147	0.842	(0.298, 4.410)
	Low liquor	0.606	0.349	3.009	0.083	1.833	(0.924, 3.636)	1.840	0.081	(0.928, 3.648)
	Beer and red wine									
Drinking frequency	Frequently	0.770	0.328	5.523	0.019	2.159	(1.136, 4.103)	2.180	0.017	(1.148, 4.140)
	Quit	−0.442	1.047	0.178	0.673	0.643	(0.083, 5.002)	0.643	0.673	(0.083, 5.004)
	Occasionally									
Amount of alcohol consumption	Binge	1.041	0.698	2.225	0.136	2.833	(0.721, 11.133)	2.850	0.133	(0.726, 11.192)
	Heavy	1.737	0.654	7.049	0.008	5.679	(1.576, 20.471)	5.756	0.007	(1.597, 20.748)
	Normal									
Physical abuse	High	0.408	0.336	1.478	0.224	1.504	(0.779, 2.904)	1.495	0.231	(0.774,2.888)
	Low									

Note: Forward stepwise logistic regression method was employed. aOR values are generated by taking into account age and education as covariance and were then adjusted for these two factors. P′ and 95%CI’are values after adjustment

**Table 7:** Multi-factor logistic regression of risk factor for alcohol dependence (*N* = 1140)

		B	SE	wald	*P*	OR	95%CI	aOR	*P′*	95%CI’
Wine category	High liquor	0.423	0.914	0.215	0.643	1.527	(0.255, 9.154)	1.526	0.644	(0.255, 9.154)
	Low liquor	1.676	0.543	9.510	0.002	5.342	(1.842, 15.495)	5.352	0.002	(1.845, 15.521)
	Beer and red wine									
Drinking frequency	Frequently	1.084	0.451	5.779	0.016	2.957	(1.222, 7.157)	2.977	0.015	(1.231, 7.202)
	Quit	0.123	1.083	0.013	0.909	1.131	(0.135, 9.450)	1.133	0.908	(0.136, 9.460)
	Occasionally									
Amount of alcohol consumption	Binge	−0.314	0.557	0.317	0.573	0.731	(0.245, 2.176)	0.734	0.578	(0.247, 2.183)
	Heavy	−0.423	0.506	0.698	0.403	0.655	(0.243, 1.767)	0.664	0.419	(0.246, 1.792)
	Normal									
Physical abuse	High	0.995	0.373	7.126	0.008	2.705	(1.303, 5.615)	2.692	0.008	(1.297,5.589)
	Low									

Note: Forward stepwise logistic regression method was employed. aOR values are generated by taking into account age and education as covariance and were then adjusted for these two factors. P′ and 95%CI’are values after adjustment

## Discussion

Extensive studies have been done to investigate the relationship between different types of trauma and alcohol consumption, which consistently demonstrated increase of alcohol consumption one year or less after trauma [25–28]. For example, a study was conducted on the substance use following terrorist attack; in a follow-up period ranging from 1 week to more than 2 years, it was found that 7.3% (95% CI: 1.1–32.5%) of the population with pre-existing alcohol use conditions reported increased alcohol consumption in the first 2 years following a terrorist event [29]. However, it is important to note that there was no new occurrences (new since the terrorist event) of alcohol use disorders observed after terrorist events [30]. Moreover, a study documented that after exposures to trauma such as floods, guns, plane crashes, etc., only 0.3% of the sample developed an acute new post-trauma alcohol use disorder [31]. Therefore, it is believed that the vast majority of post-trauma alcohol use disorders represented the continuation or recurrence of pre-existing conditions.

Long-term increases of alcohol consumption after ELS [32] has been well documented. For instance, Gondré-Lewis et al. demonstrated that ELS is as a risk factor for alcohol consumption and abuse in adulthood, such as binge drinking and impulsive-like behavior, acting through a CRF/GABA_A_ mechanism [13]. Consistent with previous studies, our study showed that there is a slightly increased prevalence of the lifetime or current alcohol use disorder in prenatal and infant exposure group compared to non-exposure group of the earthquake, although the difference among these age groups is not statistically significant. Similarly, there was no statistically significant difference in the prevalence of male alcohol use disorders for subgroups that are exposed to earthquake at different stages of pregnancy. It is important to mention that previous studies with extended follow-up periods showed that the effect of ELS on alcohol use disorder generally attenuates over time (6 months vs. 30 months) [28]. Given the fact that the assessment of alcohol use disorder was performed 38 years after the traumatic event, the attenuation effect for the present study is likely to be even more significant.

It has been increasingly evident that traumatic events in childhood contribute to subsequent psychopathology of mental health including alcohol use disorder [14, 33–36]. Most forms of child maltreatment are related to higher risk of adolescent alcohol consumption [37] and adult alcohol consumption and alcohol disorders [38, 39]. For example, Evren and co-workers have demonstrated that childhood traumatic experience, especially those related to emotional abuse, e.g., disassociation, might be a mediating factor that contributes to the development of lifetime traumatic stress disorder (PTSD) and alcohol dependence [40, 41]. Additionally, the data obtained from a study on a pair of twins showed individuals exposed to childhood maltreatment were 1.74 times more likely to experience alcohol use disorder in adulthood [42]. Bulik et al. suggested that the early life adversity may influence the development of adolescent brain in specific regions, such as slowing down the development of the hippocampus [43]. Among the early life adversities, our study showed that the childhood physical abuse (OR = 2.310, 95% CI: 1.026, 5.201) is one of the risk factors for male alcohol dependence. The physical abuse in childhood may lead to emotion regulation difficulties, which may play a significant role in alcohol use disorder [44]. With respect to the childhood sexual abuse, our study showed that there does not appear to be a strong correlation between childhood sexual abuse and alcohol use disorder in adulthood. Moreover, Bulik’s study on adult female twins also indicated no correlation between childhood sexual abuse and alcohol use disorders [43]. In contrast, another study based on retrospective assessment of childhood sexual abuse showed significantly increased chance of alcohol disorders [45]. The discrepancy on the role of early age sexual abuse plays in adulthood alcohol use is probably attributed to the difference in the selection of samples. For our study, this is also likely because childhood sexual abuse was rare in China in the 1970s.

The present work provides a comprehensive analysis on the effect of single traumatic event on individuals from different age groups and sheds light on the underlying risk factors for adulthood alcohol use disorder. However, since this study is conducted on a retrospective and self-assessment basis and a dynamic evaluation of the individual’s alcohol use shortly after trauma is lacking. It was difficult to obtain a baseline of the individual’s predisposition to alcohol use disorders 38 years after the earthquake. Further, as the prevalence of female alcohol dependence in the general population of China is only 0.09% [46], and there are fewer females in this study (about 50 in each group), we only analyzed data of male subjects. Future work employing a larger sample size for the investigation of women’s alcohol dependence after trauma is well underway.

## Conclusion

Our results suggest traumatic experience during infant and prenatal periods does not have a strong correlation with alcohol use disorders for male adults. Additionally, exposure to traumatic event during different stages of pregnancy does not affect the likelihood of adulthood alcohol use disorder. Furthermore, we identified that the consumption of low concentration liquor and frequent drinking, as well as childhood physical abuse are risk factors for alcohol dependence in male adults. The present study provides valuable insights into the correlation between alcohol use disorder and childhood trauma, and may offer guidance for alcohol use disorder therapy.

## Data Availability

The datasets generated and analyzed during the current study are available from the corresponding author on reasonable request.

## Abbreviations

CTQ-SF: The childhood trauma questionnaire short form
ELS: Early life stress
HPA: Hypothalamuspituitary-adrenal
LTE-Q: Lifetime of Experience Questionnaire
MS: Maternal separation
SCID: Structured Clinical Interviews for DSM-IV Axis Disorders (patient version)
SD: Standard deviation
